# A Case Series of Vaping-Induced Lung Injury in a Community Hospital Setting

**DOI:** 10.1155/2020/9631916

**Published:** 2020-01-31

**Authors:** Mohammed Ali, Kashmala Khan, Mihir Buch, Manuel Ramos- Ramirez, Munish Sharma, Seema Patel, Saiara Choudhury, Humayun Anjum, Alamgir Khan, Salim Surani

**Affiliations:** ^1^Department of Pulmonary Medicine, Corpus Christi Medical Center, Texas, USA; ^2^Department of Internal Medicine, Corpus Christi Medical Center, Texas, USA; ^3^Department of Pulmonary, Critical Care & Sleep Medicine, Texas A&M University, Health Science Center, Texas, USA

## Abstract

Acute and subacute injury to the lung parenchyma can be caused by multiple products. Over the past few years, vaping (also known as E-cigarettes) has become a popular trend and has been considered “safer” alternative to smoking cigarettes, especially among young adults. The use of E-cigarettes has rapidly increased, and according to the most recent report by CDC released at the end of December 2019, 2,506 cases and more than 54 associated deaths due to vaping/E-cigarette-associated lung injury were reported. Though vitamin E acetate and tetrahydrocannabinol (THC) have been found in most of the bronchoalveolar lavage samples, there are still small numbers of cases that have not reported to using THC-containing compounds. Research looking into other possible constituents in E-cigarettes that can account for the etiology of disease and effects of vaping as it relates to pulmonary physiology still remains limited and uncertain. We hereby present a case series of 5 patients who were admitted primarily for respiratory symptoms of cough, dyspnea, and fevers and were diagnosed with vaping-induced pulmonary injury.

## 1. Introduction

“Vaping is safer than smoking traditional cigarettes,” according to the literature that seems to be the rationale behind the steady rise in the use of E-cigarettes [1]. The average users are young adults who either use vaping or E-cigarettes as a bridge to quit smoking cigarettes, or they believe that they are making the healthier and smarter choice [2]. Vaping was introduced in the United States (US) market in 2007 [3]. It has gained popularity over the past few years, with celebrities and Instagram influencers endorsing its use and making it a preferred choice than regular cigarettes. E-cigarettes containing nicotine and other substances, for example marijuana, vaporizes or produces aerosols, which are then inhaled by the user. There are many flavors available in the vaping cartridges, which are particularly appealing to the younger users. Both traditional and E-cigarettes use nicotine, and some researchers have shown vaping to be just as addictive as smoking traditional cigarettes [4]. The extent of potential harmful effects of vaping is still not fully understood and is an area of much interest with public health researchers and physicians. We hereby present the 5 cases of vaping-induced lung injury, which we encountered during the very early phase of this disease process labeling.

## 2. Case 1

**Figure 1:**
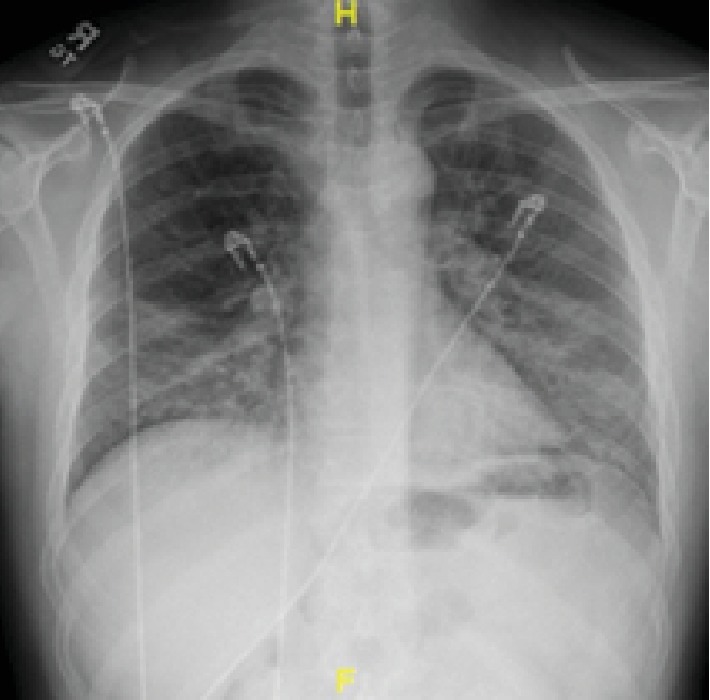
Initial chest X-ray showing bilateral patchy infiltrates with lower lobe predominance.

**Figure 2:**
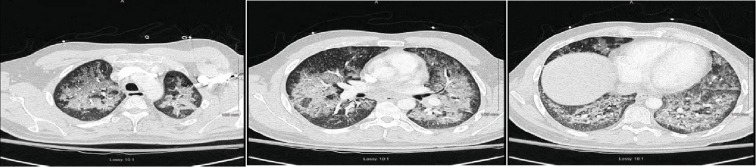
Chest CT showing bilateral airspace disease.

A 34-year-old male without any significant past medical history presented with the complaint of shortness of breath, fever, chills, and cough with mucoid sputum for a 6-day duration. He has initially been evaluated at an emergency department (ED) 2 days prior and was discharged home with the diagnosis of pneumonia on oral levofloxacin. His oxygen saturation on presentation to our ED was 84% on room air. He required 15 L/minute of oxygen via a nonbreather mask to maintain saturations over 90%. The initial arterial blood gas (ABG) revealed a pH of 7.47, PO_2_ of 41 mmHg, and PCO_2_ of 30.8 mmHg. His initial white cell count was 15.6 × 10^3^ cells/*μ*L (range: 4000-11000). Chest X-ray showed bilateral patchy infiltrates and consolidation with lower lobe predominance (Figure 1). Computed tomography (CT) with contrast of the chest was negative for pulmonary embolism but showed extensive bilateral airspace disease (Figure 2). Bronchoscopy revealed a moderate amount of thick mucoid secretions bilaterally with diffuse bronchial mucosal hyperemia.

**Figure 3:**
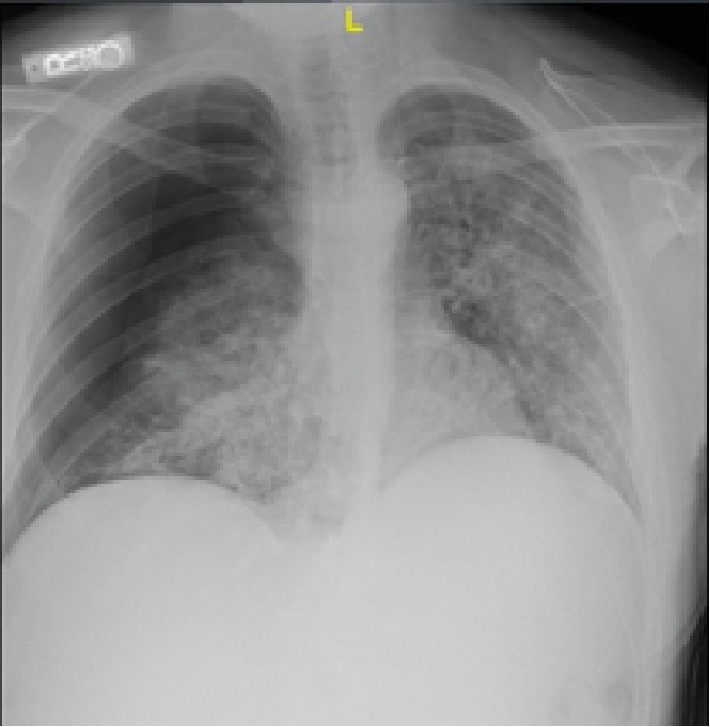
Chest X-ray showing bilateral parenchymal infiltrates and right-sided pneumothorax.

**Figure 4:**
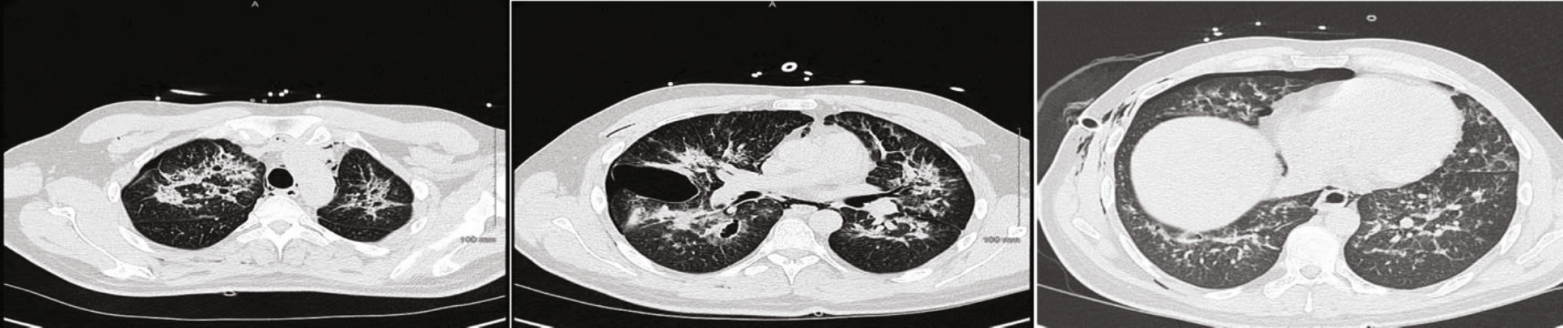
Chest CT showing a large bulla in the right lung adjacent to the major fissure with a few other bullae seen in the superior segment of the right lower lobe, pneumomediastinum, subcutaneous emphysema, and significant interstitial lung disease.

Bronchoalveolar lavage (BAL) fluid analysis revealed increased WBC with lymphocytic predominance. Fluid culture did not grow any organisms, and cytology was negative for malignancy. Clinical improvement was seen after starting intravenous methylprednisolone 40 mg every 12 hours, ceftriaxone, and azithromycin. Antibiotics were discontinued once bronchoscopy cultures did not show any organisms. An echocardiogram showed a left ventricular ejection fraction of 55-60%. He was switched to oral prednisone 50 mg daily with significant improvement in his symptoms and oxygen requirements. He was subsequently discharged on a tapering dose of oral prednisone. On the fourth day after discharge, the patient presented to the ED once again with a complaint of sudden onset shortness of breath, right-sided chest pain, and cough productive of white sputum. Chest X-ray in the ED showed a right-sided tension pneumothorax along with bilateral parenchymal infiltrates (Figure 3). Emergent needle decompression was performed, and a small bore 12 French chest tube was inserted in the ED. The CT of the chest also revealed a right-sided pneumothorax, pneumomediastinum, right-sided subcutaneous emphysema, and parenchymal disease similar to previous imaging (Figure 4). The chest tube was removed on day 4. Repeat CT chest showed no pneumothorax, but a large bulla in the right lung adjacent to the major fissure with a few other bullae seen in the superior segment of the right lower lobe, pneumomediastinum, subcutaneous emphysema, and significant interstitial lung disease. The patient underwent blebectomy via video-assisted thoracic surgery. He did well and was discharged home and was doing well on room air on outpatient follow-up in one week.

## 3. Case 2

A 22-year-old male with a past medical history of substance abuse (marijuana vape pens daily), depression, and a history of suicidal ideation presented with a fever of 101°F and productive cough with white phlegm for 2 weeks. His symptoms were associated with nausea, vomiting, nonbloody diarrhea, night sweats, and headache. His blood pressure was 123/69 mmHg; heart rate 87 beats per minute; and oxygen saturation 93% on room air.

**Figure 5:**
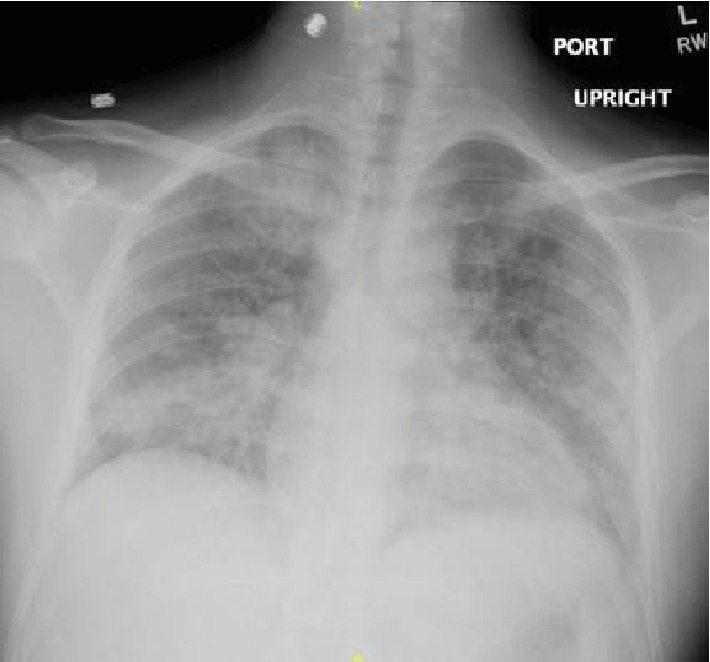
Chest X-ray showing bilateral infiltrates.

**Figure 6:**
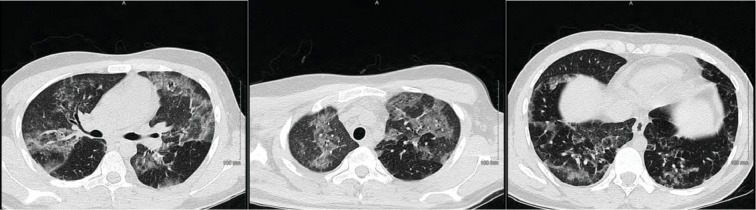
Chest CT showing bilateral scattered ground glass opacities.

During the initial course in his hospitalization, he required 6 L of oxygen via a nasal cannula. His oxygen saturation began to decline, and he required 15 L of oxygen via an oxymask to maintain his oxygen saturation above 92%. Lung examination revealed wheezing bilaterally, while other systemic examinations remained unremarkable. He had white blood cell count of 14.31 × 10^3^ cells/*μ*L, with a neutrophil predominance of 94.3%, hemoglobin of 10 gm/dL, and platelet count of 487,000 × 10^3^/*μ*. ABG revealed pH 7.46, pCO_2_ 34.0 mmHg, pO_2_ 61.3 mmHg, and HCO_3_ 22.3. Chest X-ray showed bilateral airspace disease (Figure 5) and CT of the chest revealed scattered ground glass opacity in both lungs (Figure 6). We speculated the possibility of pneumonia versus pneumonitis caused by vaping. The patient was started on ceftriaxone, doxycycline, and oral prednisone at 60 mg daily. Bronchoscopy revealed inflammatory changes in bilateral upper lobes, more so on the right than the left. Bronchoalveolar lavage (BAL) sample analysis showed acute and chronic inflammatory cells with lymphocyte predominance and rare eosinophils. Cultures from BAL did not grow any organisms. Cytology did not reveal malignancy. His viral, fungal, and bacterial studies were negative. The patient improved and decided to leave against medical advice. However, upon reaching home, he had worsening dyspnea and subsequent syncopal episode prompting another visit to the ED. He was admitted once again and treated for both pneumonia and vaping-induced lung pneumonitis. He was restarted on prednisone 40 mg daily that was tapered over the next 2 weeks. The patient's symptoms completely resolved during follow-up in 2 weeks.

## 4. Case 3

**Figure 7:**
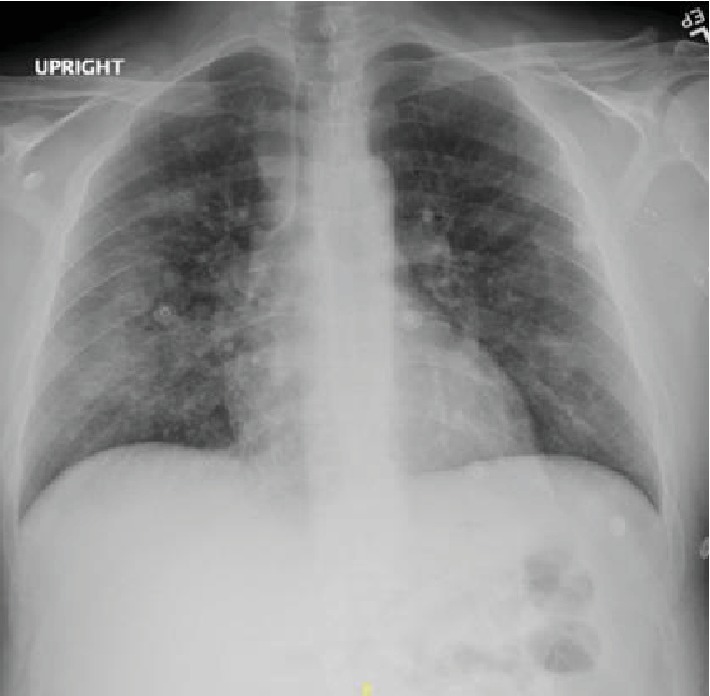
Chest X-ray showing diffuse bilateral infiltrates, more pronounced on the right side.

**Figure 8:**
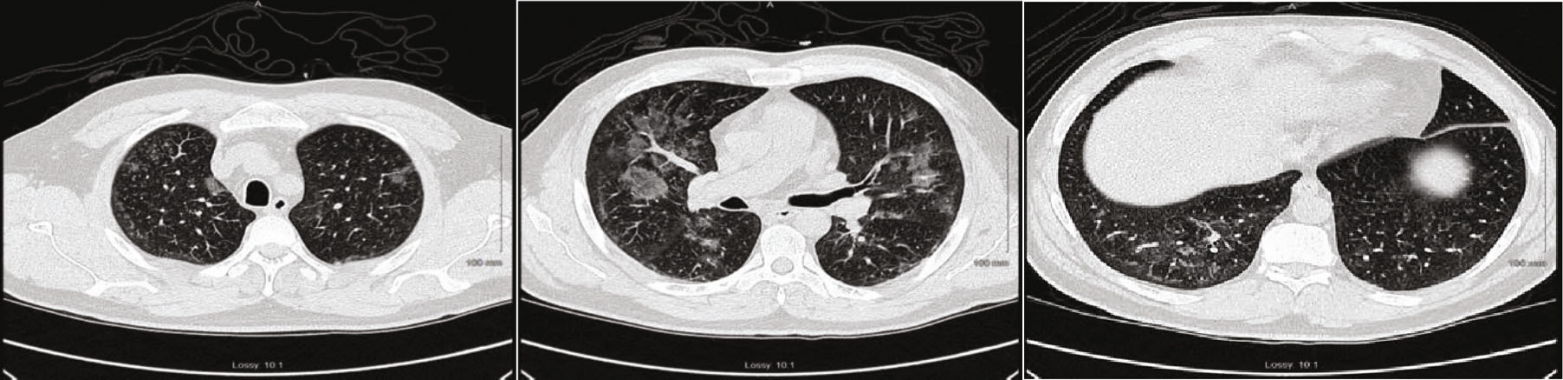
Chest CT showing nonspecific bilateral ground glass opacities.

A 42-year-old male with a past medical history of type 2 diabetes mellitus, hypertension, dyslipidemia, end-state renal disease on renal replacement therapy, and tobacco and marijuana use presented with an 8-day history of fever, cough productive of yellow sputum, myalgia, generalized weakness, multiple episodes of nausea, emesis, nonbloody diarrhea, and poor appetite. He was seen by his primary care physician two days prior to presentation in our ED and was prescribed levofloxacin. In the ED, the patient's white blood cell count was 13.0 × 10^3^ cells/*μ*L; chest X-ray showed diffuse bilateral infiltrates more pronounced on the right without pleural effusion (Figure 7). A CT chest without contrast was done which showed bilateral nonspecific ground glass opacity throughout the lungs without any pleural effusion or pneumothorax (Figure 8). Bronchoscopy showed mild clear secretions bilaterally. BAL washings had a hazy appearance with BAL fluid showing WBC 118, RBC of 3655, and 42 lymphocytes. Cultures and cytology were negative. Influenza A/B testing, human immunodeficiency virus (HIV) screen, fungal antibodies, autoimmune workup including antinuclear antibody, rheumatoid factor, anti-Ro antibody, anti-La antibody, c- ANCA, anti-Jo antibody, and anti-Smith antibody were all negative. He was started on IV methylprednisolone 40 mg every 12 hours for vaping-induced pneumonitis. The patient's symptoms significantly improved with the resolution of weakness, cough, nausea, and emesis. He was subsequently discharged on prednisone 40 mg daily tapered over 2 weeks and counseled heavily on discontinuing the use of E-cigarettes.

## 5. Case 4

**Figure 9:**
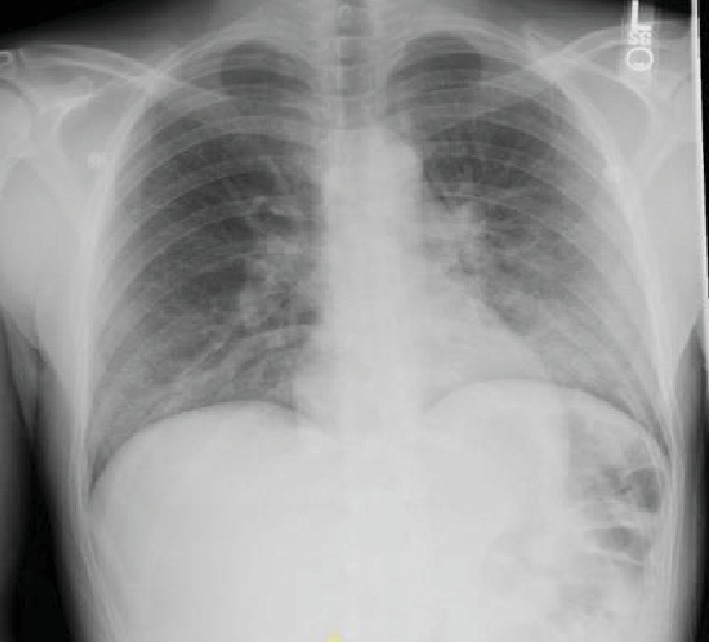
Chest X-ray showing bilateral airspace opacities.

**Figure 10:**
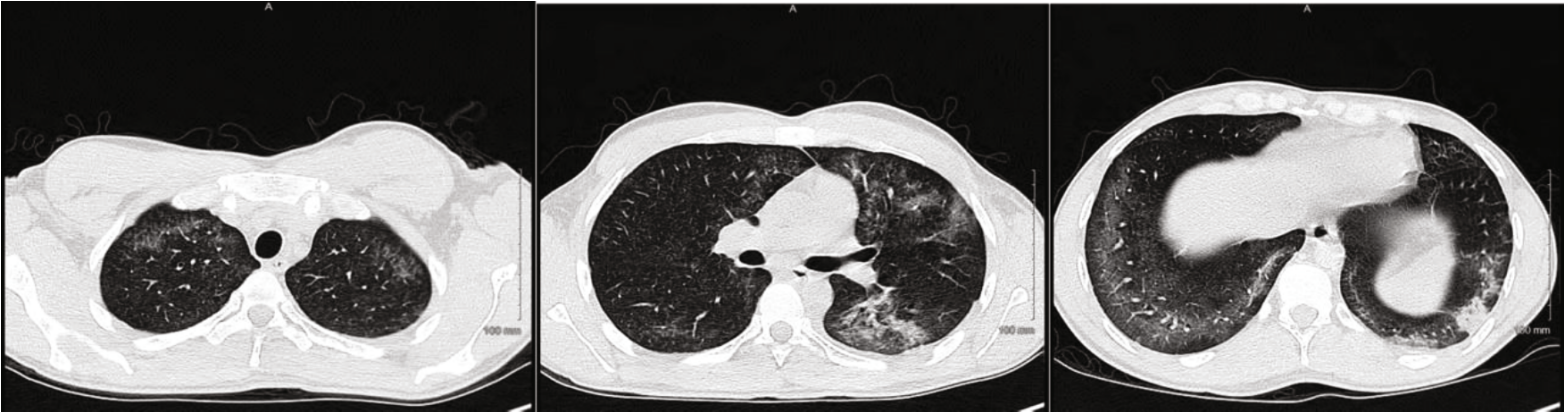
Chest CT showing bilateral diffuse infiltrates.

A 27-year-old male with no significant past medical history who presented with the complaints of shortness of breath, nausea, vomiting, and night sweats for 5 days. He reported using an E-cigarette for 6 months. He was using products containing both nicotine and tetrahydrocannabinol (THC). He stated that he had started using a “black market” cartridge containing THC 2 days before symptom onset. His vital signs showed a temperature of 100°F, heart rate of 115 beats per minute, blood pressure of 137/81 mmHg, and oxygen saturation of 98% on room air. Laboratory workup was significant for a WBC of 18.28 × 10^3^ cells/*μ*L. Chest X-ray showed airspace opacities bilaterally (Figure 9). He was started on intravenous ceftriaxone and azithromycin and admitted to the hospital. He continued to have dyspnea; therefore, a CT chest without contrast was obtained which revealed diffuse infiltrates bilaterally (Figure 10). He was then initiated on IV methylprednisolone 40 mg every day. His symptoms continued to improve during the short hospital course, and after significant clinical improvement, he was discharged home on a prednisone 40 mg daily tapered over 2 weeks. He was counseled to avoid using E-cigarettes.

## 6. Case 5

**Figure 11:**
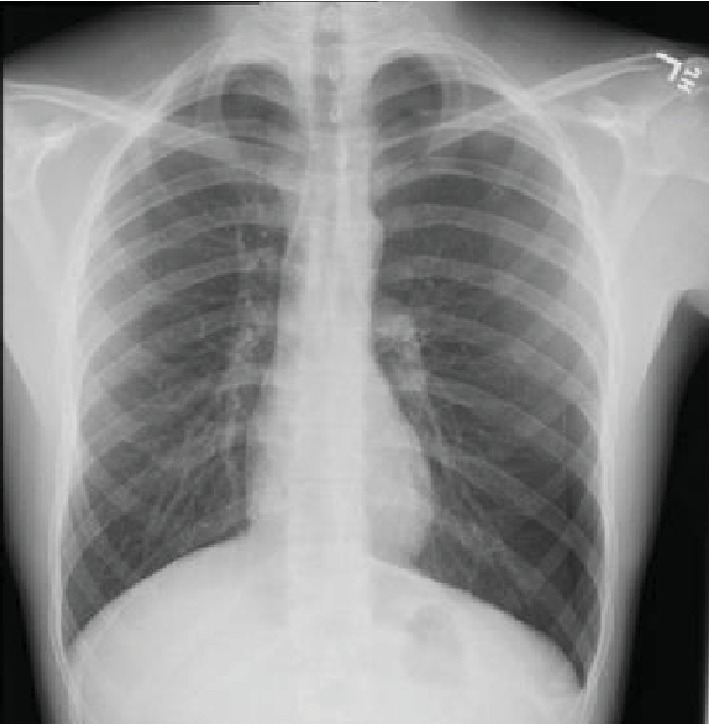
Chest X-ray showing moderate sized left-sided pneumothorax.

**Figure 12:**
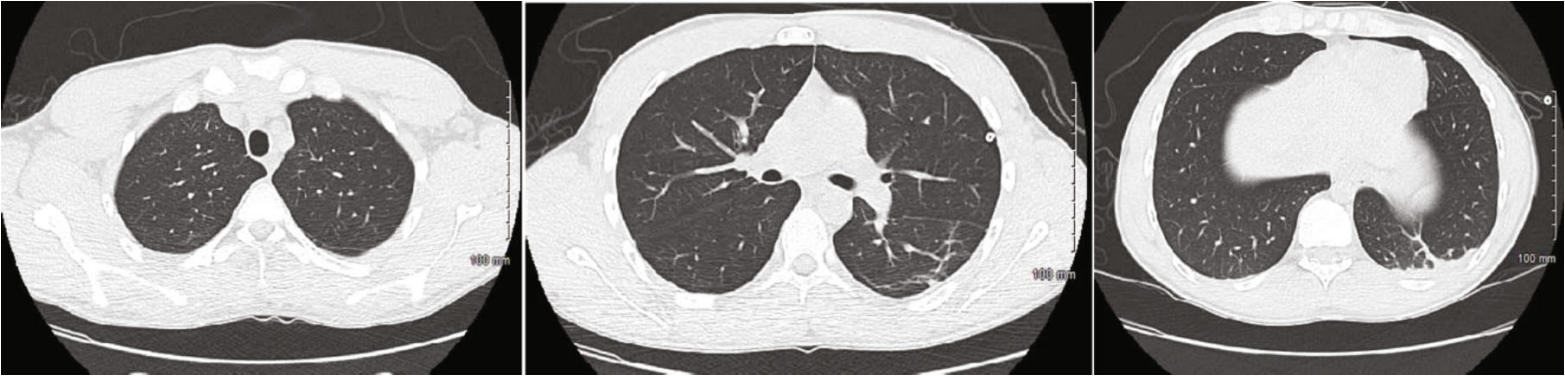
Chest CT showing left-sided atelectasis.

A 21-year-old male with no past medical history presented with acute onset of left-sided chest pain. He stated he was at work and during his break was using an E-cigarette when he developed severe left-sided chest pain and shortness of breath. He reported using an E- cigarette for the past 3 years. He stated that he only used nicotine-containing cartridges and had never used any other cartridges containing THC. He went to an urgent care center where a chest X-ray was obtained which revealed a small left-sided pneumothorax. In the ED, a repeat chest X-ray was obtained which revealed a moderate sized pneumothorax (Figure 11). A left-sided chest tube was inserted. Laboratory workup was insignificant and did not reveal any abnormal findings. CT chest was obtained which revealed left-sided atelectasis (Figure 12). His pneumothorax resolved, and the chest tube was removed on hospital day 3 and he was discharged home with instructions to avoid vaping.

## 7. Discussion

The use of E-cigarettes or vaping has risen exponentially within the past few years. Although never officially approved by the FDA, there is a common misconception that their use is safe [5]. The majority of users are teens and young adults, which makes this latest phenomenon concerning. Popularity can be attributed to aggressive manufacturer campaigns promoting these products and a sizable number of celebrities frequently seen using these products. Introduced to the US market in 2007, E-cigarettes visually mimic conventional cigarettes; however, they do not burn tobacco [3, 6]. Instead, they are battery-operated and vaporize an “E-liquid” at high temperatures producing aerosols. This liquid mixture contains nicotine and various compounds. The chemical composition of these liquids does vary, which includes, but not limited to, glycerin, propylene glycol, formaldehyde, acetaldehyde, acrolein, and various tobacco-specific nitrosamines. Propylene glycol and glycerin constitute about 66% and 24% of the liquid, respectively. One major factor at play is the numerous flavors that are available and readily added to the E-liquid.

They range from fruity flavors to unusual flavors like chocolate, coffee, etc. that are particularly appealing to the younger population [6, 7]. Recent reports of significant pulmonary illness from the use of vaping products have received significant attention. However, healthcare professionals remain at a disadvantage because there is a lack of complete understanding of how these products induce injury and cause significant symptoms.

According to the CDC, in 2015, among older E-cigarette users (aged 45 or older), 1.3% had never used traditional cigarettes. While in younger users (aged 18-24), 40% had never smoked traditional cigarettes. In 2018, more than 3.6 million US middle and high school students used E-cigarettes within the past 30 days [8]. In 2019, the CDC has reported 1299 cases, 70% of those were male, the median age was 24, and 80% of patients were under 35 years of age. On December 17, 2019, CDC published an updated document stating that 2,506 cases and more than 54 associated deaths due to vaping/E-cigarette-associated lung injury were reported [9]. We expect this data to change with the rising incidence of this disease. Initial data shows that most patients use E-cigarettes containing THC; some use nicotine while others used a combination of both nicotine and THC. Vitamin E acetate and THC were implicated as the most likely causative agents in the report released by CDC in December of 2019. Vitamin E acetate was detected in BAL samples of 48 of 51 samples collected by CDC. In this context, it is sensible to recommend avoidance of THC-containing vapes that are most likely laced with vitamin E acetate. But there are still a persistent small number of cases that have not reported the use of THC-containing compounds [9]. The FDA is analyzing samples collected from patients across the country that have suffered from this illness and is testing a variety of chemicals including but not limited to THC and nicotine. Interestingly, though vitamin E acetate and THC have been found to be the most likely culprit, no single chemical has been solely linked to these reports. We think that there might be more compounds playing the causative role that are yet to be unearthed. There is also scarcity of a definite clinical practice guideline in terms of the evaluation and management of the vaping-induced lung injury [10].

These case series of 5 patients that we presented were recently hospitalized with similar symptomology. These patients presented to the hospital between July and September of 2019, which encompass the early phase of this epidemic. They had symptoms of dyspnea, nausea, and vomiting as the most common presenting symptoms. Imaging showed diffuse bilateral ground glass opacities except in case 5, which predominantly showed pneumothorax only. Further understanding of the pathophysiology of vaping-induced lung injury is needed. To date, no absolute histopathologic findings have been linked to the use of E-cigarettes. In addition, BAL findings were nonspecific. With vaping being introduced almost a decade ago, the question that naturally arises is what changed? There is no conclusive evidence as to why so many people have suffered over the past few months. Studies need to be conducted to better understand the root cause of this challenge. Per our assessment, there must have been either an environmental change, change in the E-cigarette, or its components to have caused this serious epidemic. Another factors to take into consideration are genetic factors or common phenotype among the affected individuals. There are some limitations, which were noted in this case series. Not all patients underwent bronchoscopy, and therefore, we do not have BAL results. Lung biopsies were not done because they were not warranted at the time as the patients began to improve clinically. The treatment was not standardized as this was an emerging condition and the data on treatment was nonexistent.

## 8. Conclusion

We have presented the case series of 5 patients who were seen in the earlier stage of this disease emergence. Since our submission and revision process, the number of patients with this condition has drastically increased and the pathophysiology seems to have become little more apparent. We, as authors, still feel that there is much more to be learned in this condition, and we may not be surprised if pathophysiology and the inciting and exacerbating factors change over time as more patient data and information emerge. We feel that these early case reports will entice more vigilance among the readership to have an open mind as they come across the clinical vignette and urge them to be more proactive in reporting and studying this disease. It is prudent to continue reporting all the cases of vaping-induced lung injury and its subsequent complications in a relentless manner. Further data and evidence emanating from every possible stratum of the healthcare community will play a vital role in zeroing down on all the harmful compounds in the E-cigarettes. In the wake of recent data by CDC pertaining to vitamin E acetate and THC, we may be tempted to rest our case prematurely.

## References

[1] Moore K., Young H., Ryan M. F. (2015). Bilateral pneumonia and pleural effusions subsequent to electronic cigarette use. *Open Journal of Emergency Medicine*.

[2] Caponnetto P., Campagna D., Cibella F. (2014). Correction: Efficiency and safety of an electronic cigarette (ECLAT) as tobacco cigarettes substitute: a prospective 12-Month randomized control design study. *PLoS One*.

[3] Layden J. E., Ghinai I., Pray I. (2019). Pulmonary illness related to E-cigarette use in Illinois and Wisconsin — preliminary report. *The New England Journal of Medicine*.

[4] Yamin C. K., Bitton A., Bates D. W. (2010). E-cigarettes: a rapidly growing internet phenomenon. *Annals of Internal Medicine*.

[5] Etter J. F., Bullen C., Flouris A. D., Laugesen M., Eissenberg T. (2011). Electronic nicotine delivery systems: a research agenda. *Tobacco Control*.

[6] Grana R., Benowitz N., Glantz S. A. (2014). E-Cigarettes. *Circulation*.

[7] Pellegrino R. M., Tinghino B., Mangiaracina G. (2012). Electronic cigarettes: an evaluation of exposure to chemicals and fine particulate matter (PM). *Annali di Igiene*.

[8] (2019). Electronic cigarettes fact sheet. http://www.CDC.gov/.

[9] https://www.cdc.gov/media/releases/2019/t1220_telebriefing_update_lung_injury.html

[10] Kalininskiy A., Bach C. T., Nacca N. E. (2019). E-cigarette, or vaping, product use associated lung injury (EVALI): case series and diagnostic approach. *The Lancet Respiratory Medicine*.

